# Developing a Sustainable Need-Based Pediatric Acute Care Training Curriculum in Solomon Islands

**DOI:** 10.3389/fpubh.2017.00086

**Published:** 2017-04-24

**Authors:** Daniel Ta Yo Yu, Jason T. Gillon, Raymond Dickson, Karen A. Schneider, Martha W. Stevens

**Affiliations:** ^1^Department of Pediatrics, Johns Hopkins Hospital, Baltimore, MD, USA; ^2^National Referral Hospital, Honiara, Solomon Islands; ^3^Pediatric Emergency Department, Bloomberg Children’s Center, Baltimore, MD, USA

**Keywords:** curriculum design, train-the-trainer, Solomon Islands, pediatric acute care, global health medical education, ADDIE model

## Abstract

**Background:**

The Johns Hopkins Hospital Pediatric Emergency Department (PED) was invited to collaborate with the National Referral Hospital (NRH), Solomon Islands, to establish an acute care pediatric education program for the country’s inaugural class of national medical graduate trainees.

**Objective:**

To develop and evaluate a sustainable, need-based post-graduate training curriculum in pediatric acute care, resuscitation, and point-of-care ultrasound.

**Methods:**

A need-based training curriculum was developed utilizing the ADDIE model and was implemented and revised over the course of 2 years and two site visits. Implementation followed a train-the-trainer model. The curriculum consisted of high-yield didactics including workshops, simulations, hands-on ultrasound sessions, and lectures at the NRH. A mixed-methods design was used to evaluate the curriculum, including pre/posttesting, qualitative group discussions, and individual surveys. The curriculum was revised in response to ongoing learner evaluations and needs assessments. Continuing educational sessions after the site visit demonstrated sustainability.

**Results:**

The curriculum included 19 core topics with 42 teaching sessions during the two site visits. A total of 135 pre/posttests and 366 individual surveys were collected from 46 trainees. Completion rates were 78.2% for surveys and 71.3% for pre/posttests. Pre/posttest scores increased from 44 to 63% during the first site visit and 69.6 to 77.6% during the second. Learners reported a mean 4.81/5 on a standard Likert scale for curriculum satisfaction. Group discussions and surveys highlighted key areas of knowledge growth, important clinical care advances, and identified further needs. Initial sustainability was demonstrated by continued ultrasound sessions led by local graduate trainees.

**Conclusion:**

A collaborative team including Johns Hopkins PED staff, Solomon Islands’ graduate trainees, and NRH administration initiated a professional education curriculum for the first class of Solomon Islands’ medical graduates. Knowledge growth and positive impacts of the program were reflected in learner survey and test scores. Graduate trainees were identified as local champions to continue as course instructors. This innovative curriculum was developed, revised, and initially sustained on site. It has been successful in introducing life-saving pediatric acute care and graduate training in Solomon Islands.

## Introduction

One of the most difficult elements for establishing a functional health-care system for a low middle income country such as the Solomon Islands is having a sufficient supply of locally based trained health-care workers (1). Solomon Islands, located in the South Pacific, is comprised of six major islands among more than 900 smaller islands (1–4). With 70% of the population living in remote or very remote areas, access to medical care in Solomon Islands is complicated by the long travel distances and dispersed islands (1).

In 2007, Solomon Islands established a new health aid program with Cuba, which provides consistent education for Solomon Islands medical students every year. In 2014, the first 22 newly graduated doctors arrived home facing the challenge of practicing medicine with limited resources and a shortage of further structured graduate medical training. With the support of the Embassy of Taiwan in Solomon Islands and a Solomon Islands-based medical team from Kaohsiung Medical University Hospital in Taiwan, the Johns Hopkins Hospital (JHH) Pediatric Emergency Department (PED) has collaborated with the National Referral Hospital (NRH) to establish a training curriculum with a heavy focus on pediatric acute care clinical education for these new doctors.

The NRH is the largest hospital and the only referral hospital in Solomon Islands. It is located in the capital city of Honiara on the island of Guadalcanal. The NRH offered a pediatric emergency team from Johns Hopkins the opportunity to provide medical education to the inaugural class of graduate trainees with particular emphasis on the acute care of critically ill children. Teaching consisted of high-yield didactics in the form of workshops, simulations, and lectures. The curriculum was designed based on the ADDIE method (analysis, development, design, implementation, and evaluation) of instructional systems design, with the curriculum content developed according to real-time needs assessment. In addition to teaching basic hands-on pediatric resuscitation skills and pediatric acute care topics, a primary goal was to establish a curriculum that could be repeated by local instructors at the NRH after completion of a site visit.

The ADDIE model is a popular instructional design process used in creating a teaching curriculum that is geared toward producing specific learning outcomes and behavioral changes (5). It provides a systematic approach to the analysis of learning needs, the design and development of a curriculum, and the implementation and initial evaluation of a training program (5, 6). This model of developing new training programs is particularly useful if the focus of the program is targeted toward changing participant behavior and improving performance (6, 7). While it was first widely used by the US Armed Forces for technical training and flight training in the 1970s (8), recent studies have successfully adopted the ADDIE model to improve patient safety, procedural competency, and disaster simulation (7, 9, 10). It has also been effectively used in medical training to change practice behaviors in the management of various medical conditions (11–13). While frequently cited, it is important to note that the ADDIE model does not appear to have an original author and authoritative definition. Its application has evolved informally through each individual author’s interpretation (14, 15). This flexible yet organized approach in creating relevant focused learning outcomes was an ideal roadmap for the curriculum design process.

## Objectives

This project was conducted to provide need-based pediatrics training to the Solomon Islands medical graduate trainees. Using the ADDIE model, we aimed to develop a training curriculum in acute pediatric care, resuscitation, and point-of-care ultrasound. We aimed to implement the curriculum utilizing a train-the-trainer model and evaluate the impact of the curriculum, including knowledge growth, learner experience, satisfaction, future needs, and sustainability.

## Methods

A mixed-methods design was used to develop, implement, and revise a curriculum over the course of 2 years and two site visits. The study protocol received JHH Institutional Review Board exemption, approval from the NRH, and all participants consented to surveys and interviews.

## Setting

Over the course of two 4-week site visits in September 2015 and September 2016, a training program was developed and implemented at the NRH of Solomon Islands. The participants were local health-care practitioners who were required to manage a wide range of medical complaints with limited resources and direction. The vast majority of participants were medical graduates of Solomon Island citizenship who completed their medical school education in Cuba with the remaining minority graduating from regional medical schools in Fiji or Papua New Guinea. All local practitioners were fluent in English as well as Pijin, the nation’s lingua franca; however, the inaugural class of residents who graduated from medical school in Cuba were further faced with the challenge of translating their medical knowledge from Spanish into their local dialects and English.

## Project Design

**Figure 1:**
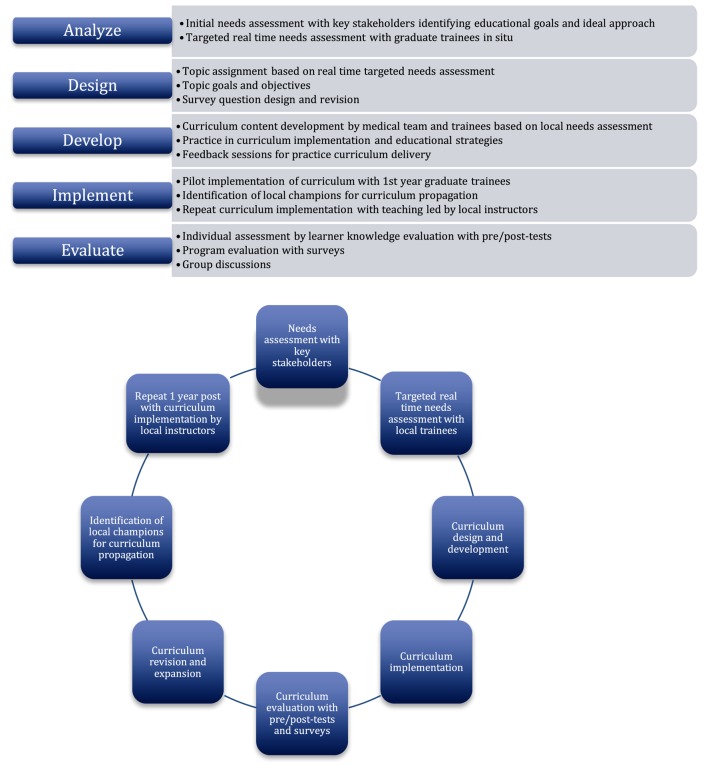
**ADDIE model and project design**.

The design process of the pediatric acute care curriculum consisted of an adaptation of the five phases of the ADDIE model (Figure 1), beginning with identifying trainee needs, educational goals, and optimal methods of content delivery. This was followed by implementation and evaluation of the resulting curriculum and learner performance. Finally, emphasis was placed on curriculum maintenance and sustainability by employing a train-the-trainer model in the second curriculum implementation phase. Although the ADDIE model phases are written in a linear order, its application is often considered to be iterative and cyclical (14). Concepts from Kern’s Six-Step Approach to Curriculum Development and Medical Education were applied to the ADDIE model (16). Kern’s six-step approach consists of (i) problem identification and general needs assessment, (ii) targeted needs assessment, (iii) goals and objectives, (iv) educational strategies, (v) implementation, and (vi) feedback and evaluation (16). Kern’s approach placed particular emphasis on a non-linear progression of curriculum development process. While the ADDIE model appeared more user friendly and was the primary framework of curricular design of this project, a more dynamic approach was used with each step contributing to changes and improvement of the final product.

## Analysis

An initial needs assessment was conducted by interviewing key collaborators including the NRH Superintendent, Head of Pediatrics and Hospital Education Coordinator. These administrators were invited by email to identify graduate educational needs and specific equipment availability. This preliminary needs assessment guided development of a preliminary curriculum prior to the first site visit. We were also assisted by the Embassy of Taiwan in Solomon Islands and a locally based medical team from Kaohsiung Medical University Hospital to establish local government, hospital leadership, and media connection. Government and leadership recognition not only permitted smooth passage through customs control with large quantities of medical supplies but also helped facilitate our complex conference scheduling of a resulting total of 42 formal teaching sessions.

Once on site, we formally met with the key educational stakeholders, the graduate trainees, to discuss the preliminary curriculum and identify the most relevant needs in pediatric acute care instruction for an initial curriculum. After each educational session with the trainees, we obtained immediate feedback with qualitative surveys and group discussion to assess the educational impact of that session and identify shortcomings. This feedback was directly applied to the design of future sessions, allowing for continuous quality improvement of our curriculum.

## Design

During initial immediate feedback sessions, the trainees identified several high-yield topics for further instruction. These real-time feedback sessions not only informed the development of overarching goals and objectives for these topics but also allowed for ongoing modification of the overall educational curriculum.

Post-session surveys were designed to assess learner experience with open-ended questions and 5-point Likert scale responses. We adapted Floyd and Fowlers reference book on Improving Survey Questions (17). We followed a quality control checklist for each survey question and revisions were based on feedback from a pilot group of five physicians in the JHH pediatric emergency medicine division.

## Development

The curriculum development team consisted of three groups: the JHH group, the NRH administration, and Solomon Islands’ graduate trainees. The JHH team consisted of medical volunteers from the PED, including a pediatric emergency medicine attending and fellow, pediatric residents, and a pediatric emergency nurse. The NRH administrative team consisted of the Head of Pediatrics, the Medical Superintendent of the NRH, and the Hospital Education Coordinator. The graduate trainees group was previously described in Section “Setting.” Curriculum development was iterative and ongoing throughout the 2 years, and sustainability measures are ongoing currently.

Teaching consisted of high-yield didactics in the form of workshops, simulations, hands-on ultrasound sessions, and lectures at the NRH. Different topics, depending on the learning objective and available material, required different delivery strategies and content preparation. The curriculum was practiced and prepared according to available resources. Teaching feedback to graduate trainee instructor was provided by the JHH pediatric emergency medicine fellow and attending.

## Implementation

The pilot implementation phase during the first visit in September 2015 targeted the inaugural class of Solomon Island medical graduates who predominantly obtained their medical degrees in Cuba. During the second site visit in September 2016, an expanded training curriculum utilizing a train-the-trainer model was implemented, targeting a new class of medical graduates in addition to the class taught the year prior.

Local volunteer instructors from the second-year group were identified prior to the second site visit in order to take over responsibility of the curriculum. Formal ongoing feedback was provided for local instructor-led sessions. Due to high level of interest and positive survey response, particular focus was placed on point-of-care ultrasound during the second site visit.

## Evaluation

A mixed-methods approach was used to evaluate the curriculum, which consisted of pre/post-event knowledge testing as well as post-event qualitative group discussion and individual qualitative surveys. Participant knowledge growth was evaluated from scores from pre/posttesting of each session. Test questions written by the pediatric emergency fellow and attending covered key learning objectives from the education sessions. Pre/posttesting of sessions during the first and second site visits were identical but questions were presented in a different order.

The post-session group discussion and individual qualitative surveys reflected the impact of the curriculum, learner experience, and learner satisfaction. Group discussions were conducted with focused questions assessing what was learned, what could be improved, applicability of practice with available resources, confidence in teaching the same topic again independently, and other topics to include in the future. The curriculum was further revised in response to the first site visit evaluations and needs assessments. Educational sessions that were led by local instructors after the site visit were used to evaluate sustainability.

## Results

The curriculum was delivered to a total of 57 participants. Average age of participants was 29.3 years old. A total of 46 participants were graduate trainees who completed medical school abroad. Two graduated from medical school in Fiji and two graduated from medical school in Papua New Guinea. A total of 42 graduated from medical school in Cuba; 20 were first-year trainees that graduated in 2015 and 22 were second-year trainees that graduated in 2014. Other participants included six visiting medical students from Australia and five local nurses.

**Figure 2:**
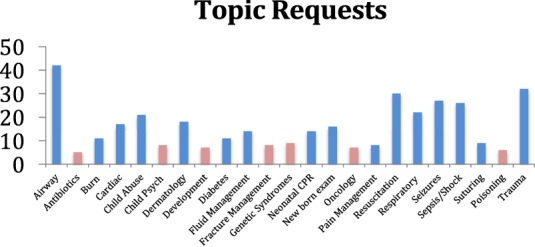
**The topics with the most requests (highlighted in blue) were incorporated into the curriculum**.

Through qualitative surveys, graduate trainees identified topics of greatest interest. Figure 2 shows the topics that were requested by individual trainees and the number of times each topic was requested. The curriculum was tailored to cover the most requested topics (in blue). Topics with less than three requests were not listed. Even though ultrasound teaching was not initially requested by trainee surveys, a special bedside ultrasound curriculum was requested by key hospital stakeholders (NRH Superintendent, Head of Pediatrics and Hospital Education Coordinator) in the general needs assessment prior to the second site visit.

The resulting curriculum consisted of 16 core topics delivered during two site visits comprising a total of 42 teaching sessions. These included early newborn care, drowning, seizures, sepsis, pediatric/neonatal resuscitation, airway management, common rashes, suturing, respiratory emergencies, diabetes, child abuse, pain management, cardiac murmurs, point-of-care ultrasound, and trauma (Table 1).

**Table 1:** **Curriculum content and delivery**.

Workshops	Simulations	Lectures
Basic airway	Trauma	Early newborn care
Suturing	Sepsis	Drowning
FAST ultrasound	Pediatric resuscitation	Seizures
Cardiac ultrasound	Neonatal resuscitation	Shock
Lung ultrasound		Common rashes
		Respiratory emergencies
		Diabetes
		Child abuse
		Pain management
		Cardiac murmurs

Our curriculum was evaluated using three methodologies: post-event qualitative group discussions, individual surveys, and pre/post-event knowledge testing. Group discussions occurred after all 42 sessions. Individual surveys were collected after 15 of 42 sessions. Pre/post-event knowledge testing was completed after selected resuscitation topics (trauma, sepsis and shock, and airway management). Completion rates are shown in Table 2.

**Table 2:** **Survey and testing completion rates**.

Site visit	366 surveys	135 pre/post tests
	*n* (% complete)	*n* (% complete)
2015^a^	167 (84.3)	57 (86.3)
2016^b^	199 (72.1)	78 (56.3)

*^a^22 trainees*.

*^b^46 trainees*.

Overall knowledge gains were reflected by a pre/posttest score rise from 44 to 63% during the first site visit and 69.6 to 77.6% during the second site visit. The first site visit group consisted of 22 trainees that graduated in 2014. The second site visit group consisted of 46 trainees, which included the initial group of 22 trainees from the first site visit. Due to test anonymity, individual participant progression was unable to be tracked.

Learners reported a mean 4.81/5 (5 = very satisfied) on a standard 5-point Likert scale for curriculum satisfaction and 4.5/5 (5 = very relevant) for relevance of teaching session to current practice. Upon self-assessment, learners reported a mean 3.3/5 (5 = understands topic very well) for understanding of topic before the teaching session and 4.1/5 for understanding of topic after the teaching session. Learners reported 3.85/5 (5 = very confident) for confidence in teaching the same topic again if given access to teaching supplies and materials.

## Qualitative Feedback and Group Discussion

Focus group discussions and surveys highlighted key areas of knowledge growth and important clinical care advances. The evaluation process focused upon what was learned, what could be improved, and other topics to be included in the future.

When asked about practice changes, feedback included: “I learned about the different ways to give oxygen,” “I learned how to correctly use a defibrillator,” and “I learned that oral salbutamol is not as effective as inhaled salbutamol.” Participant response also reflected increased confidence in managing pain, seizures, and caring for infants: “I feel comfortable with the practice of pain management and using different types of analgesics for mild to severe pain,” “Excellent presentation, I feel more confident in managing children with seizures, especially febrile seizures,” and “I feel more prepared looking after newborn infants in the first min after birth.”

Child abuse was a particularly well-received topic based on survey feedback. The majority of trainees reported no prior formal instruction of this topic. Participants wrote: “We really need a good reporting system for child abuse, today’s topic really got me thinking the victim are people who can’t speak for themselves and it is up to me to see that the help they need is provided,” “Thank you so much for this valuable session, I feel more confident in recognizing child abuse. I think this condition is always ignored here,” and “This is very valuable in our daily practice.”

When asked about recommendations for changes and improvement, most responses indicted the need to provide more education—more topics, more time per session, and more repeat sessions. Many requested video format of the sessions (18 responses). Feedback also revealed that the teachers were speaking too fast (14 responses). As the ADDIE model was applied in a dynamic iterative manner, the response to this feedback could be made in real time. Subsequent survey comments demonstrated improvement in lesson delivery after speaking at a slower pace (three responses).

When asked open-ended questions about teaching–learning strategies and which aspects they found new or useful, answers were overwhelmingly positive about demonstrative teaching methods combined with PowerPoint slides. Representative responses included: “I’ve never seen actual demonstrations of the standard approach to assessing trauma patients and also practice them directly after.” Another wrote: “I learned a new approach of teaching by using slides combined with practical demonstrations” and “The seizure management demonstrations were wonderful.”

## Sustaining the Curriculum

The maintenance and enhancement of the curriculum relied on collaborative efforts. The Johns Hopkins team and the NRH administration were responsible for the initial development of the curriculum content, but it was through the active participation, input, and feedback from graduate trainees that the curriculum was tailored to be need-based and the preferred content delivery was established. This ensured appropriate high-yield teaching topics that were pertinent to the graduate trainees’ level of knowledge, local practices, and available resources. This in turn likely promoted the trainees’ high level of interest and engagement. This is thought to have helped with bridging the cultural gap as the local Polynesian and Melanesian culture have been observed as shy and reserved when “exposed” to Western culture, which can be initially misinterpreted as being disinterested to the outsider.

Curriculum material including airway supplies, simulation manikins, referencing resources, and the complete series of PowerPoint lectures were left behind for local use. Two local volunteer instructors were selected from the second-year group to take over responsibility of the curriculum. Formal feedback was provided for local instructor-led point-of-care ultrasound sessions. An additional three hands-on ultrasound sessions were led by a local volunteer instructor showing initial evidence for sustainability. Periodic emails with the local instructor helped to maintain ownership and responsibility. The program was featured three times by Solomon Islands’ local news agency, with one story in particular highlighting the local volunteer instructor demonstrating ultrasound use. This further added to local stakeholder satisfaction of the program and in turn can motivate this local curriculum champion to further expand on the curriculum.

The Hopkins team plan to maintain contact to continue to support and evaluate program needs.

## Discussion

The training program was well received and demonstrated further generalizability of the ADDIE model. This was the first formal post-graduate training program implemented in Solomon Islands for pediatric acute care.

The “analysis” component of the ADDIE model, in which daily lectures and/or simulations were designed to address the specific topics requested by local trainees, was critical to learner attendance. While numerous lectures and simulations were prepared in advance based on needs identified by the hospital leadership, lectures on topics such as diabetes and seizure management were identified from learner request, implemented into the lecture schedule, and highly attended. This adaptability proved quite popular, and end-of-lecture negotiation about the following day’s topic resulted in sustained attendance of the majority of the trainees.

The mixed teaching methods consisting of PowerPoint lectures, simulations, and workshops received very positive feedback but could have been easily modified within those parameters if a trainee learning preference was identified. While we were able to bring six pediatric-sized mannequins and a wide array of airway equipment for simulations, other simulation materials were obtained from local resources, including banana peels for mock flesh for a suturing workshop and empty plastic juice bottles as mock neonatal mannequins for neonatal resuscitation simulation. While higher fidelity simulations are preferable, pretest and-posttest scores were comparably improved between simulations regardless of materials used. Taché et al. used inner tubes from bicycle tires as mock small bowel and plastic tubes covered with foam and vinyl as mock saphenous veins beneath flesh to teach small bowel repair and venous cutdown among other procedures to senior medical students at a teaching hospital in Tanzania (18). They found through objective structured clinical examination (OSCE) scores a fourfold improvement in skill compared to pre-training OSCE scores with large effect sizes, demonstrating utility of low-cost, low-fidelity simulations.

The largest limitation of effective implementation of the ADDIE model was effective curriculum propagation after the first site visit. It was difficult to pre-identify local champions of the curriculum nor was there sufficient time to repeat the curriculum with teaching led by local instructors. After first-year trainees had been exposed to the curriculum, it was much easier recruiting interested now-second-year trainees to teach part of the curriculum during the second site visit. Depending on time constraints, it is likely that at least two site visits will be required to recruit local curriculum champions as the first site visit foremost served as an opportunity not only to develop the curriculum, but more critically to develop friendship and trust between medical teams and foster an academic partnership. A second site visit confirmed ongoing commitment to the host institution, and the local instructors we hoped to recruit—the second-year graduate trainees—were already familiar with our team and generally knew what to expect.

Two second-year graduate trainees were identified as curriculum champions and given the opportunity to lead various lectures, workshops, and simulations. At 3-month follow-up, one local volunteer champion had taught cardiac and lung ultrasound a total of three times and had been featured in the local newspaper teaching a workshop. This workshop was most likely in demand as it is skill based, utilizes an important technology in a very resource-limited setting, and had received strongly positive survey feedback. It is likely impromptu ultrasound training sessions were easier to set up compared to simulations and lectures, which tend to require greater organization. Compared to the other teaching topics that were not technology dependent, this one unfortunately relies on the sole ultrasound machine continuing to function.

Another potential limitation of curriculum propagation was the lack of repetition of the curriculum. Identified local trainees received no more than two sessions per topic separated by 1 year. The overall demonstrated increase in pre/posttest knowledge score averages from the two site visits, along with the positive qualitative survey responses does not necessarily translate into achieving competency in performance. It is likely that even with intensive feedback given to the second-year graduate trainees after their training sessions during the second site visit, they may not feel adequately trained to autonomously lead teaching sessions without support. The train-the-trainer approach was hybridized with primary curriculum delivery given time constraints, and more formal and separate instructor training sessions may have led to further success in curriculum propagation. It is well documented, particularly in regards to cardiopulmonary resuscitation, that brief but frequent “booster” training is important for skill retention (19).

Since time was limited, it may have been more beneficial to focus on a smaller selection of the most requested topics, such as airway and ultrasound, allowing ample time for repetition and local trainee teaching practice. Malan et al. (11) were able to implement a widely adopted motivational interviewing program for primary care providers in the Western Cape of South Africa through use of the ADDIE model. Factors likely contributing to their success include a focused curriculum related to a defined topic (motivational interviewing) and formal train-the-trainer instruction in which trainers were taught the curriculum with the explicit expectation that they would propagate it. While the variety of lectures was appreciated and likely contributed to high learner attendance and participation, the capacity for curriculum propagation and long-term reinforcement was compromised.

As a result of this experience, it is our impression that the ADDIE model is a useful training curriculum implementation tool targeted toward medical graduate trainees in Solomon Islands. Cuba, where the vast majority of trainees obtain medical degrees, has very different social determinants of health, available resources, and the sociocultural relationship to Western medicine is quite different. The ADDIE model served as a way to integrate sociocultural nuances into the curriculum since curriculum design is directly based on a real-time needs assessment. As an example, when a lecture on child abuse during the first site visit was being presented, attendees mentioned that pediatric burn patients are frequently encountered because open fires, particularly for trash burning, are commonplace, and many were curious from their own experiences about distinguishing accidental from non-accidental burns. Consequently, the child abuse lecture during the second site visit was largely focused specifically on burn patterns and burn management.

In future site visits, our inter-hospital team will continue to apply the ADDIE model consistently to demonstrate long-term validation of the model in Solomon Islands. Future goals include promoting the successful propagation of the ultrasound workshop and teach additional targeted applications of point-of-care ultrasound such as obtaining peripheral intravenous access and assessing cardiac function. It has been shown in rural Rwanda that ultrasound teaching is sustainable, particularly with ongoing quality assurance by review of emailed key images to instructors between site visits, identification of ultrasound champions to sustain enthusiasm, and ultrasound machines that are maximally durable to resist the elements (20). Finally, we will restrict the remaining curriculum to fewer topics determined from real-time targeted needs assessment to leave ample time for both knowledge or skill reinforcement and intensive local champion teaching practice and feedback. We will also be persistent with establishing ongoing dialog with identified local champions after future site visits to help encourage curriculum propagation and identify solutions to any barriers.

## Conclusion

Based on our experience, we believe that the ADDIE model is an efficient and effective way to develop a local professional education curriculum in Solomon Islands. The curriculum was successfully implemented for the first class of Solomon Islands’ medical graduates. Knowledge growth and positive impacts of the program were reflected in survey and test scores. Local champions were identified to continue as course instructors and efforts are in place to sustain the curriculum and continue future collaboration. Future education endeavors will focus on a similar model with particular emphasis placed on more frequent booster training for instructor-led sessions on a smaller selection of topics to further enhance sustainability. The curriculum should be adapted to local resources available at the time of a site visit and continually refined to optimize sociocultural relevancy.

## Ethics Statement

The JHM IRB has determined that the above-referenced study (IRB00081486) qualifies as exempt research under the DHHS regulations 45 CFR 46.101(b)(1). Research conducted in established or commonly accepted educational settings, involving normal educational practices, such as (i) research on regular and special education instructional strategies, or (ii) research on the effectiveness of or the comparison among instructional techniques, curricula, or classroom management methods.

## Author Contributions

DY (Johns Hopkins Hospital) was primary involved in the overall design and development stages of the project. The rest of the training sessions, data collection, and evaluation of feedback was mostly done by DY, JG and RD and overseen by KS and MS. All the authors contributed to the article and approved the final manuscript.

## Conflict of Interest Statement

The authors declare that the research was conducted in the absence of any commercial or financial relationships that could be construed as a potential conflict of interest.
